# Silica- and Titanium-poly(ethylene glycol) Hydrogels—Novel Matrices for Bacterial Cell Immobilization

**DOI:** 10.3390/gels11110934

**Published:** 2025-11-20

**Authors:** Ekaterina Filippova, Anton Zvonarev, Vasily Terentyev, Vasilina Farofonova, Valeriya Frolova, Tat’yana Khonina, Sergey Alferov, Daria Lavrova

**Affiliations:** 1 Laboratory of Ecological and Medical Biotechnology, Tula State University, Lenin Pr. 92, 300012 Tula, Russia; katya.filippova010197@gmail.com (E.F.);; 2 FSBIS FRC Pushchino Scientific Centre of Biological Research, G.K. Skryabin, Institute of Biochemistry and Physiology of Microorganisms, Russian Academy of Sciences, Nauki Pr. 5, 142290 Pushchino, Russia; zvonarevibpm@gmail.com; 3 FSBIS FRC Pushchino Scientific Centre of Biological Research, Institute of Basic Biological Problems, Russian Academy of Sciences, Institutskaya St. 2, 142290 Pushchino, Russia; 4 FSBIS FRC Pushchino Scientific Centre of Biological Research, Institute for Biological Instrumentation, Russian Academy of Sciences, Institutskaya St. 7, 142290 Pushchino, Russia; 5 I.Ya. Postovsky Institute of Organic Synthesis, S. Kovalevskoy St. 22, 620990 Yekaterinburg, Russia

**Keywords:** silica-polyethylene glycol, titanium-polyethylene glycol, hydrogel, bacteria immobilization, sol–gel, biohybrid materials, biomimetics

## Abstract

For the first time, hydrogels based on silica- and titanium-poly(ethylene glycol) have been used for immobilization of Gram-negative bacteria (*Escherichia coli MG1655*) and Gram-positive bacteria (*Rhodococcus qingshengii X5*) in a one-step sol–gel synthesis. Vibrational spectroscopy and thermogravimetric analysis have confirmed the formation of amorphous hybrid structures with a predominance of organic components and metal-oxide grids. Encapsulation efficiencies were 72–77% for Si-PEG-based hydrogel and 50–54% for Ti-PEG. Antimicrobial activity tests revealed that Si-PEG was non-toxic, while Ti-PEG reduced cell viability by 50%. For the first time, an analysis of the morphological properties of immobilized bacterial cells revealed the formation of a thin Si-PEG-based hydrogel shell around each cell and a thick polymer layer on the bacterial surface when encapsulated within Ti-PEG-based hydrogels. The catalytic activity of the biocatalysts, as measured by the ATP content, remained at 84–93% for Si-PEG-based hydrogel, and decreased to 5% for Ti-PEG-based hydrogel. Biocatalysts based on encapsulated bacteria in a Si-PEG-based hydrogel demonstrate high sensitivity and stability. Si-PEG-based hydrogel exhibits high biocompatibility, making it suitable for the effective encapsulation of various bacterial types with a “cell-in-shell” structure.

## 1. Introduction

Gels based on natural and synthetic polymers, such as alginate [1,2], agar, poly(ethylene glycol) (PEG), poly(vinyl alcohol) (PVA) [3], silica gels [4], and polyacrylamide, are widely used to immobilize various enzymes and cells in order to produce valuable biocatalysts [5,6]. These gels have a three-dimensional porous structure, which not only allows for the retention of biomaterials but also ensures effective mass transfer, allowing substrates and reaction products to diffuse freely [7]. An important advantage is the ability to precisely control the properties of the gel matrix, such as porosity, mechanical strength, and hydrophilicity, in order to optimize the immobilization conditions for various microorganisms [8]. Despite the biocompatibility of these hydrogels, natural polymers are biodegradable. This means that their structure will deform over time, which can be a significant disadvantage when it comes to their industrial use.

Sol–gel technology has long been considered an affordable and convenient way to stabilize biocatalysts [9,10,11,12,13,14]. However, there are several limitations of the sol–gels for whole cell immobilization, including the release of alcohol, shrinkage during drying, and the use of a far from neutral pH for sol–gel synthesis. The use of acids and alkalis can also cause osmotic stress, which can negatively affect the biocompatibility of the material. To overcome these challenges, two-step sol–gel processing can be employed [15]. In the first step, the precursor is hydrolyzed at a low pH, followed by an intermediate storage stage and a neutralization-gelation step. Additionally, controlled vacuum evaporation of alcohol prior to encapsulation can be used to eliminate alcohol impact [16]. However, this requires additional resources and material costs for the industry, as well as increasing the time and complicating the process of obtaining biocatalysts. Also, during the sol–gel process using metal alkoxide, a rigid matrix forms, which can cause the cells encapsulated within it to lyse, leading to the proliferation of cells outside the matrix. To reduce this pressure and increase cell survival, structure-controlling agents, such as natural or synthetic polymers, are added to the system [17]. Promising matrices for encapsulating microorganisms are organo-inorganic hydrogels based on silicon oxide or ORMOSIL type I [18]. These hydrogels are formed by the co-polymerization of inorganic precursors such as Si-, Ti-, Zr-, and Al-alkoxides with organic components [19]. It has previously been shown that introducing organic polymers like alginate, PVA, and PEG into the classical precursor tetraethoxysilane (TEOS) used in sol–gel synthesis leads to decreased pressure on the cells, creates a conformal microenvironment around cells, and, as a consequence, causes an increase in the activity of encapsulated cells [13,20,21,22,23,24,25]. Silicon compounds in these preparations can be substituted with titanium compounds. Materials based on titanium dioxide are resistant to pH changes, are thermally stable, and have stronger mechanical strength than silica [26]. Another advantage of using titanium dioxide as a medium for capturing biomaterials is its ability to selectively absorb organophosphorus compounds, such as nucleotides [27] and phospholipids [28]. However, this approach does not completely eliminate the use of acids and alkalis in sol–gel synthesis or alcohol release, requiring the additional steps to remove them.

**Figure 1:**
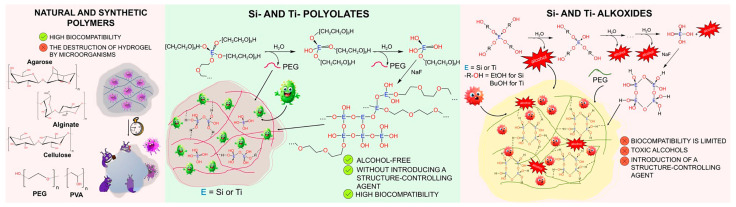
A comparison of cell encapsulation methods: the advantages of alcohol-free, one-step sol–gel synthesis using Si-/Ti-polyolates compared to traditional approaches.

Silicon and titanium polyolates combine the advantages of silicon and titanium alkoxide compounds and polymer materials (Figure 1). One of the significant advantages over traditional alkoxide materials is the possibility of a one-step immobilization of various cells. This is due to the fact that no toxic compounds are released during sol–gel synthesis, which can harm microorganisms. In addition, no extra structure-controlling agents are required, as they are formed as a by-product during sol–gel synthesis. Silicon-containing polyolate precursors (glycerol [29] and sorbitol [30] derivatives), have been successfully used to encapsulate enzymes. Previously, our scientific group [18] used silicon poly(ethylene glycol) to encapsulate whole cells of methylotrophic yeast within organosilica shells through a one-step sol–gel synthesis. It was demonstrated using biosensors that the yeast remained viable for up to 10 days and was capable of effectively degrading methanol. Based on titanium polyolate compounds, various pharmaceutical compositions have been developed for the treatment of skin, soft tissue, and mucosal membranes [31,32,33].

The encapsulation of individual bacteria within silicon and titanium polyolate-containing hydrogel matrices has not previously been systematically studied. However, the development of these new matrices for the effective immobilization of bacteria is of significant interest for biotechnology applications. The use of mild sol–gel synthesis conditions may allow for the preservation of the viability of various bacterial types, as well as ensuring effective mass transfer of nutrients and metabolic products, making these systems promising for application in biosensors, pharmaceuticals and sustainable environmental remediation technologies.

Thus, the study focused on a comprehensive analysis of hydrogels based on silicon and titanium polyethylene glycols to assess the possibility of using them for the effective immobilization of whole cells of Gram-negative *Escherichia coli* MG1655 and Gram-positive *Rhodococcus qingshengii* X5 bacteria using one-step sol–gel synthesis.

## 2. Results and Discussion

### 2.1. Structural and Functional Characteristics of Si-PEG and Ti-PEG Hydrogels

The use of vibrational spectroscopy (FTIR- and Raman spectroscopy) allows the appearance of new absorption bands to be identified in a system after sol–gel synthesis. This indirectly proves the mechanism of hydrogel formation. For Si-PEG, it has previously been shown that absorption bands from organic (PEG) and inorganic (Si–O–Si) components have been observed in IR spectra [18]. The solid phase of the hydrogel is composed of 80% organic fragments, namely PEG residues, with no crystallinity present in the solid phase [34], confirming the polymeric nature of hydrogels based on Si-PEG.

**Figure 2:**
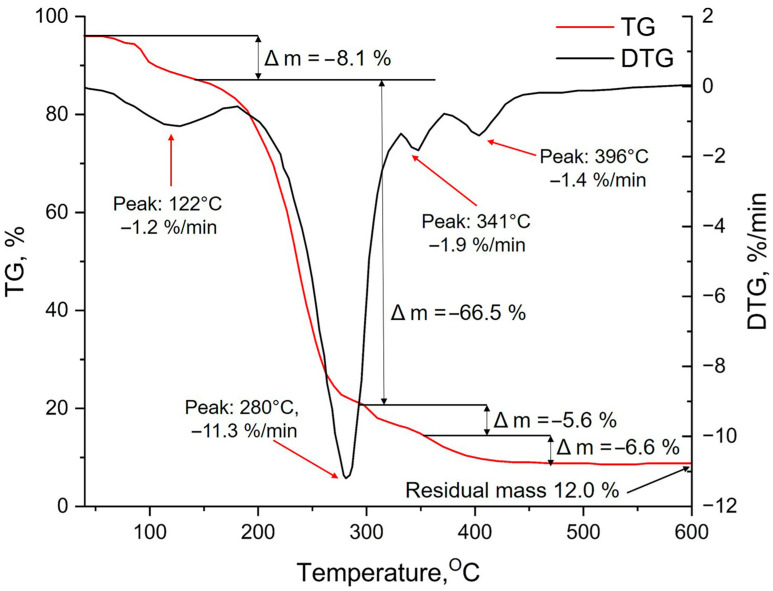
Thermogravimetric analysis of the Si-PEG-based hydrogel: curves of thermogravimetry (TG) and differential thermogravimetry (DTG).

The results of the thermogravimetric analysis of the Si-PEG-based hydrogel (Figure 2) indicate that water is the primary component during the initial stage of weight loss (peak 1 at 122 °C, with a decrease in the sample mass of 8.1%). This suggests that water is being removed from the surface, either as adsorbed water or as residual silanol (Si–OH) groups condense.

Then, at 280 °C (peak 2), the largest mass change of the sample occurs, which is associated with the removal of free PEG and bound water. When the sample is heated above 300 °C, (peaks 3 and 4), hydrated silicon oxide undergoes a metamorphic transformation and turns into a silicon-silicon oxide composite material [35], as well as the combustion of a polymer bound in a hydrogel. The residual mass of silicon dioxide is about 12%, and it is susceptible to further changes at higher temperatures.

**Figure 3:**
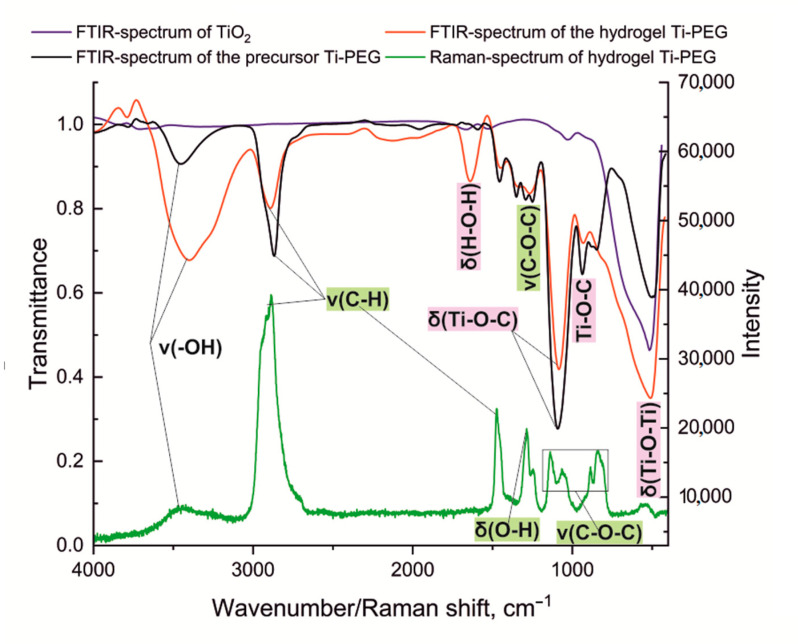
Vibrational spectra of the initial Ti-PEG, its hydrogel, and titanium dioxide.

To characterize the Ti-PEG-based hydrogel, we obtained FTIR spectra of the initial precursor TiO_2_ and the respective hydrogel, and performed a comparative analysis (Figure 3). The FTIR spectrum of the initial Ti-PEG compound shows the following bands ν/cm^−1^: 3338 (ν, –OH); 2870 (ν, C–H); 1110 (ν, C–O–C); 951; and 1110 (δ, Ti–O–C). The FTIR spectrum of the obtained hydrogel exhibits absorption bands, ν/cm^−1^: 3466 (ν, –OH); 2875 (ν, C–H); 1643 (δ, H–O–H); 1103 (ν, C–O–C); 949 (Ti–O–C_dis_); 1103 (δ, Ti–O–C); and 512 (δ, Ti–O–Ti). In the FTIR spectrum of the hydrogel, a strong absorption band corresponding to Ti–O–Ti vibrations is observed at 512 cm^−1^. The presence of absorption bands associated with PEG vibrations, along with the emergence of a new absorption band associated with titanium–oxygen vibrations, confirms the incorporation of the organic polymer into the inorganic matrix, forming a hybrid structure.

When analyzing the Raman spectrum (Figure 3, green line) of the Ti-PEG-based hydrogel, nine distinctive peaks were identified. The automatic comparison using INSPECTOR ver. 2.7.0.3704 software revealed a strong correlation (R = 0.997) with the spectrum of 3,6,9-trioxo-α-undecan-1-ol, characterized by variations in the primary alcohol group, carboxyl group, and sp^2^ hybridized carbon atom in the –CH_2_ group. The absorption band at 529 cm^−1^ has a low intensity, which can be attributed to the low content of the inorganic component in the system. This band corresponds to the deformation vibrations of the Ti–O bond, which has been previously supported by FTIR spectroscopy. The remaining absorption bands in the spectrum characterize the organic polymer material, represented by PEG-400. The absorption bands at 845 and 1141 cm^−1^ indicate valence vibrations of the C–O–C bond. The absorption band at 1289 cm^−1^ is associated with deformation vibrations of the primary alcohol group. The absorption band at 1479 cm^−1^ characterizes asymmetric valence vibrations, while the band at 2891 cm^−1^ represents symmetric valence vibrations of the CH_2_ group. The low-intensity band at 3463 cm^−1^ reflects O–H stretching vibration due to the water content of the gel. Based on the results, we can conclude that the organic component predominates over the inorganic component in the system. Besides, under the conditions of sol–gel synthesis at pH 7.0, a bridge Ti–O–Ti forms.

**Figure 4:**
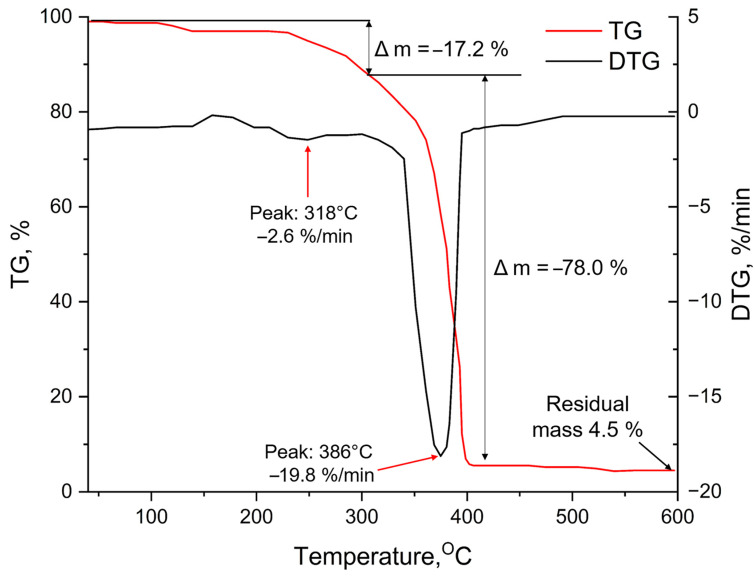
TGA of a hydrogel based on Ti-PEG.

When analyzing DTG for a Ti-PEG-based hydrogel, two stages of change in the mass of the sample were identified (Figure 4).

Peak 1 at 318 °C, with a decrease in the mass of the sample of 17.2% of its initial value, can be explained by the removal of strongly chemisorbed water by titanium cations in titanium oxohydrate (TiO_2_·x H_2_O, Lewis acid centers), as well as the initial thermal decomposition of the organic matrix, that is as a product of sol–gel synthesis. This explains the high temperatures needed to release the bound water. The process of dehydration of titanium oxohydrate to form titanium dioxide in the anatase modification, and the combustion of the hydrogel-bound polyethylene glycol, take place at 386 °C with a 78% reduction in the mass of the sample (peak 2). At 598 °C, the residual mass was 4.5%, it consists of titanium dioxide that is susceptible to changes under the influence of higher temperatures.

Thus, our findings have confirmed the formation of amorphous hybrid structures in Si-PEG and Ti-PEG systems, with a predominance of the organic component. In both cases, oxide grids (Si–O–Si or Ti–O–Ti), associated with PEG polymer chains, are formed. TGA shows the decomposition of the organic component at 280–400 °C with the formation of residual oxides (12% SiO_2_ for Si-PEG and 5% TiO_2_ for Ti-PEG). When comparing the dry residues, it can be seen that there is more polymer present in the Ti-PEG system compared to the Si-PEG one. This is because the titanium precursor used in the synthesis of Ti-PEG is obtained in excess of the polyethylene glycol, in order to prevent its intermolecular condensation. The spectroscopic data provide support for a polycondensation mechanism in the formation of the gel.

### 2.2. Photoactivity of Ti-PEG-Based Hydrogel Toward to Methylene Blue

It is well known that the photocatalytic activity of TiO_2_-based materials is directly related to their antimicrobial properties. This relationship is also closely linked to the structural and morphological characteristics of these materials [36]. The titanium dioxide phase has a significant impact on the degradation of dyes. Studies have shown that ordered crystal structures are the most active; for example, Degussa P25 nanopowder degrades dyes by 81.4% and TiO_2_ in anatase phase by 54%, while rutile phase TiO_2_ does not have any such activity [37]. In addition to this, amorphous titanium dioxide does not have any photocatalytic activity [38]. Therefore, prior to bacterial immobilization, it was necessary to determine the photocatalytic properties of a Ti-PEG-based hydrogel.

Methylene blue (MB) was selected as a model contaminant because of its well-characterized and sensitive reaction to photocatalytic degradation. This makes it an ideal tool for detecting the presence of photoactive crystalline phases in TiO_2_-based hydrogels.

**Figure 5:**
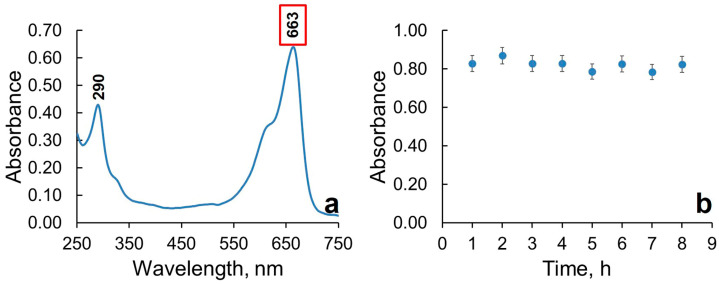
The spectral characteristics of methylene blue (**a**) and the degradation curve (**b**) of the dye in a Ti-PEG-based hydrogel.

To determine the operating wavelength, the spectral characteristics of the methylene blue solution were recorded (Figure 5a). MB absorbs at 290 and 663 nm, in the ultraviolet (UV) and visible regions of the spectrum, respectively. Based on literature data, the degradation of MB through photocatalysis with titanium dioxide [39], films [40], and particles [41] is studied at a wavelength of approximately ~660 nm [42] in the visible spectrum range. Therefore, 663 nm was chosen as the operating wavelength for the experiments. The decrease in the maximum absorbance peak at 663 nm over time can be attributed to the photocatalytic activity of the sample. This means that the sample is photodegrading the dye.

Based on the results, a graph of optical density vs. time (Figure 5b) was constructed, which shows the kinetic degradation of MB. The graph indicates that for 8 h, there were no significant changes in optical density, suggesting the absence of photocatalytic properties in the Ti-PEG-based hydrogel. In previous studies on titanium hydrogels based on polyolate precursors, it was found that crystalline structures were absent in the solid phase of the gel [43]. These results are also supported by TGA, which shows that at 385 °C, titanium dioxide transitions from an amorphous phase with no photoreactivity to an anatase form with photocatalytic activity.

### 2.3. Evaluation of the Efficacy of Bacterial Immobilization in Si-PEG- and Ti-PEG-Based Hydrogels

Gram-negative bacteria *Escherichia coli MG1655* (*E. coli MG1655*) and Gram-positive bacteria *Rhodococcus qingshengii X5* (*R. qingshengii X5*) were chosen as objects for immobilization. *E. coli MG1655* was selected due to its widespread application as a main model for genetic research [44] and its production of recombinant proteins [45]. The ability to redirect the metabolic pathways of the bacteria makes it possible to use them in the environmental purification and biofuel production. *R. qingshengii X5* is able to utilize a wide variety of organic compounds, including alkanes, aromatic hydrocarbons, and their derivatives [46]. In addition to the removal of pollutants, these bacteria are also capable of producing various secondary metabolites that have practical significance. These include antibiotics [47], carotenoids and phenylpropanoids such as curcumin A and B [48], as well as siderophores and biosurfactants [49]. The selection of *E. coli* and *R. qingshengii* is particularly important due to the differences in the structure of the cell walls of these microorganisms. Gram-negative *E. coli* has a lipopolysaccharide membrane, while Gram-positive *R. queenshengii* has a thick peptidoglycan layer with mycolic acids. These differences may affect the interaction with the hydrogel and, consequently, the morphology and functional characteristics of the final biocatalyst.

The data of the number of CFUs obtained from free and immobilized microbial cells were used to determine the effectiveness of encapsulation (see Table 1).

The encapsulation efficiency was calculated using Formula (1):

(1) $$EE,\%=\frac{\mathrm{lg}N}{\mathrm{lg}N_{0}}\times 100,$$

where N is the number of living cells released from the encapsulated hydrogel, and N_0_ is the initial number of free cells.

A comparative analysis of the results revealed that the encapsulation efficiency in Si-PEG-based hydrogel was at least 70%, and in Ti-PEG-based hydrogel, it was approximately 50%. This may be primarily attributed to the insignificant antimicrobial properties of amorphous titanium dioxide. The antimicrobial activity of Ti–O– implemented by its positively charged surface, which interacts with the negatively charged bacterial cell wall. When the surfaces come into contact, depolarization occurs, disrupting the barrier function of the membrane. Molecular targets on the membrane surface include proteins, lipopolysaccharides in Gram-negative bacteria, and lipoteichoic acids in Gram-positive bacteria [50,51]. Adhesion to the surface can cause mechanical stress and the formation of micropores in the membrane. This can disrupt the function of the sodium–potassium pump and lead to imbalances in ion concentrations, eventually causing cell death [52].

A comparative analysis of the results obtained in this study with the results described in the literature is presented in Table 2.

The results show that Si-PEG-based hydrogel has several advantages over traditional gels, such as alginate and chitosan. It provides optimal bacterial encapsulation, which is essential for wide range applications. Unlike alginate systems, which have high encapsulation efficiency (74–90%) but are susceptible to biodegradation due to bacterial enzymatic degradation [57,58], Si-PEG-based hydrogel maintains structural stability, preventing uncontrolled degradation. Chitosan, despite its good barrier properties, often reduces cell viability by 34–67% due to potential toxicity [59], whereas Si-PEG-based hydrogel is completely biocompatible. At the same time, Si-PEG-based hydrogel offers encapsulation efficiency that is comparable to alginate (72–77%) for various strains, including *E. coli* and *R. qingshengii*. It surpasses Ti-PEG-based hydrogel by 18–27%, making it a promising option for the development of long-term biocatalyst systems. Si-PEG-based hydrogel combines the best properties of both alginate (high encapsulation efficiency) and chitosan (mechanical strength), effectively eliminating their individual drawbacks.

### 2.4. Studies on the Antimicrobial Properties of Hydrogels and Their Components

Prior to immobilization, it was important to assess the effect of the initial components and their hydrogels on the bacterial cells under study. To do this, the agar diffusion method was used. The results are presented in Table 3.

For titanium compounds, cell death has been shown to be caused by the initial compound Ti-PEG and a hydrogel based on it. It should be noted that, considering the confidence intervals, the lysis zones are similar, indicating the toxic effect of these compounds on cells. It is important to note that these properties depend on the concentration of the compound used and will require additional research in the future to reduce toxic effect.

In this work, initial precursors were used without additional dilution to produce hydrogels. No lysis zones were observed for Si-PEG-based hydrogels, indicating their nontoxicity to cells and, consequently, their high biocompatibility.

**Figure 6:**
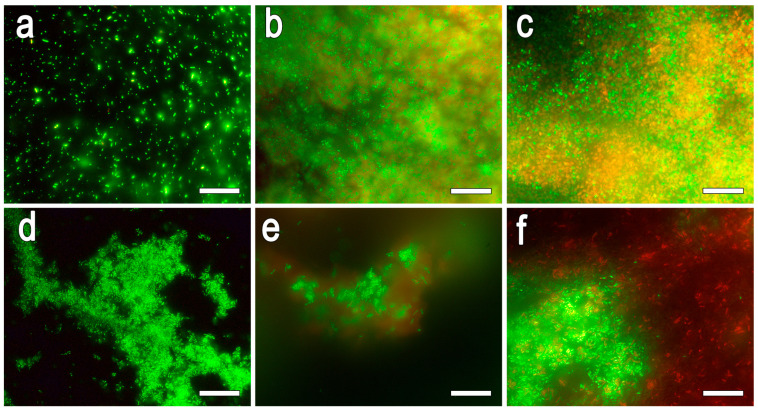
Assessment of the viability of microorganisms using the live/dead staining method: (**a**–**c**) *E. coli* MG1655 and (**d**–**f**) *R. qingshengii* X5; (**a**,**d**)—control—free cells, (**b**,**e**)—immobilization cells in Si-PEG-based hydrogel; (**c**,**f**)—immobilization cells in Ti-PEG-based hydrogel. Scale bar is 20 μm.

Fluorescence microscopy was used to identify live and dead cells before (control) and after immobilization in hydrogels (Figure 6).

When free microorganisms are stained, the cells are alive and fluoresce green. The survival rate of bacterial biomass in Ti-PEG-based hydrogel was approximately 50% of the initial value. Additionally, it is worth noting that the hydrogel does not accumulate dye on its own, which could indicate the presence of large pores where the dye does not linger and instead penetrates into the cells. After encapsulation in the Si-PEG-based hydrogel, the survival rates of *E. coli* (Gram−) and *R. qingshengii* (Gram+) were found to be similar, which may be attributed to the loss of viable cells during encapsulation. However, for *R. qingshengii*, the toxic effects of the components may have been less pronounced due to its thicker cell wall, which can retain low-toxic compounds. Additionally, the turbidity on the images may be explained by the absorption of light by the hydrogel.

A combination of methods, including evaluation of encapsulation efficiency, assessment of component toxicity using agar diffusion, and analysis of cell viability through fluorescence microscopy, has shown that the Si-PEG-based hydrogel is non-toxic and offers high encapsulation efficiency (72–77%). In contrast, the Ti-PEG-based hydrogel exhibits antimicrobial activity, resulting in a decrease in cell survival and reduction in encapsulation efficiency to approximately 50%. Therefore, the Si-PEG matrix offers significant advantages for creating viable and functional biocatalysts based on whole cells. However, it is this combination of properties—the lack of photocatalytic activity in the inorganic matrix (Ti–O–) and the predominance of the biocompatible organic phase (PEG)—that opens up new possibilities for Ti-PEG hydrogels. We believe that this system could be an excellent basis for enzyme immobilization.

### 2.5. Investigation of the Morphology of Biohybrid Materials—Immobilized Bacteria in Hydrogels

**Figure 7:**
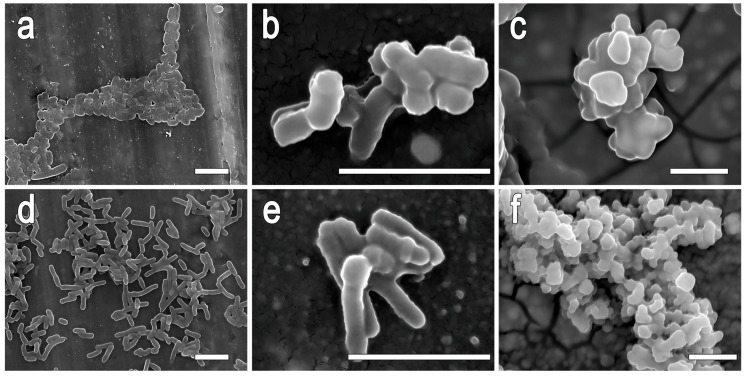
SEM-images of the free cells and biohybrid materials—immobilized bacteria in hydrogels: (**a**–**c**) *E. coli* MG1655, (**d**–**f**)—*R. qingshengii* X5; (**a**,**d**)—control—free cells, (**b**,**e**)—immobilization cells in Si-PEG-based hydrogel; (**c**,**f**)—immobilization cells in Ti-PEG-based hydrogel. Scale bar is 5 μm.

The morphology of free cells and immobilized bacteria in hydrogels based on silicon and titanium polyolates was studied using scanning electron microscopy (SEM) (Figure 7).

When using silicon derivatives, microbial cells are covered with sol particles over the entire surface, creating a heterogeneity in the material in the form of “waves” and “elevations” (Figure 7b,e). This allows the microorganisms to be distinguished from each other and indicates that the layer of hydrogel formed around the cells is much thinner compared to a similar titanium compound. Additionally, a “cell-in-shell” material is formed. When using titanium precursors, a thick polymer hydrogel layer forms on the cell surface (Figure 7c,f). This has been confirmed by the increase in cell size of *E. coli* and *R. qingshengii*, which has grown from 0.8 microns to 3 microns in width. Sol particles do not form on the cell surface due to the strong predominance of the organic polymer component over the inorganic component in the system.

**Figure 8:**
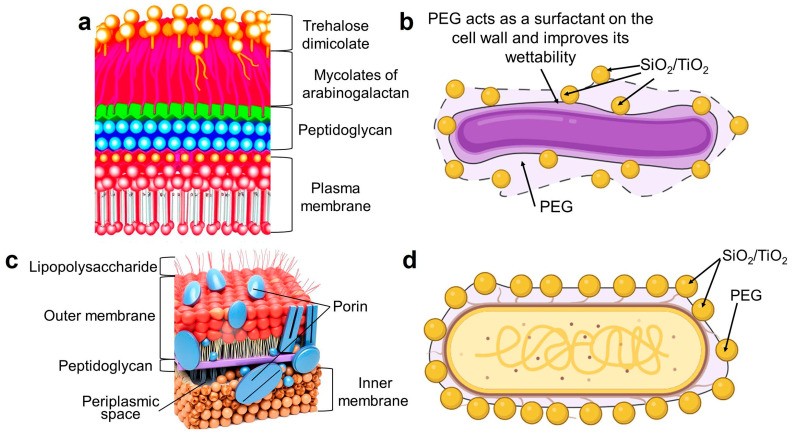
The proposed mechanism of a hydrogel shell formation around bacterial cells: (**a**) the structure of the cell wall of Gram-positive (actinobacteria); (**b**) the shell around *R. qingshengii* X5; (**c**) the structure of the cell wall of Gram-negative bacteria; (**d**) the shell around *E. coli* MG1655.

The structural components of the cell surface play a crucial role in the formation of organo-inorganic shells around microorganisms. This process is illustrated in Figure 8, which shows the proposed mechanism of shells formation.

When studying the natural processes of silicate formation in diatoms, it has been found that modifying the amino acid residues of certain silicate peptides that participate in this process, along with long-chain polyamines, can change the structure of silica during the sol–gel process from tetramethoxysilane (TMOS) in vitro [60]. It is assumed that the formation of this shell occurs in two stages: nucleation and growth. At the physiological pH of Gram-negative *E. coli*, the negatively charged lipopolysaccharide groups on the cell surface weakly interact with Si(OH)_3_^−^ and Ti(OH)_3_^−^. This slows down the nucleation process, but PEG acts as a “glue”, compensating for this interaction between the cell and Si–O– or Ti–O–. As a result, a spongy mesoporous structure with high regularity forms around the cell. For *R. qingshengii*, poly(ethylene glycol)can act as a surfactant, improving the wettability of the cell wall. After that, hydrophobic mycolic acids bind with silicic and titanic acid residues more efficiently, forming an intermittent coating with SiO_2_-PEG and TiO_2_-PEG clusters.

### 2.6. Catalytic Activity of Encapsulated Bacteria in Hydrogels

**Figure 9:**
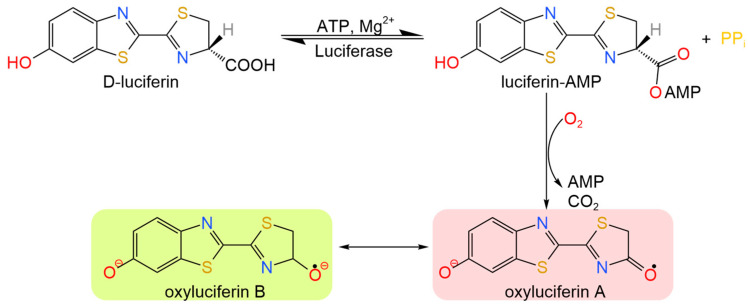
Bioluminescent reaction scheme for ATP determination. The bioluminescent reaction involves the oxidation of luciferin by ATP, which is catalyzed by the enzyme luciferase in the presence of Mg^2+^.

The catalytic activity of microorganisms was determined by measuring the content of adenosine triphosphate (ATP) in a sample and by using biosensor technique. The method is based on a classic bioluminescence reaction, where ATP (which was extracted from cells through lysis), in the presence of the luciferase enzyme and Mg^2+^ catalyzes the oxidation of luciferin. This process produces a light signal whose intensity is proportional to the concentration of ATP in the sample (Figure 9). This technique allows for the qualitative or quantitative (if calibrated) determination of the number of microorganisms’ cells, as the intensity of the glow is proportional to the concentration of ATP molecules involved in the reaction [61,62].

**Figure 10:**
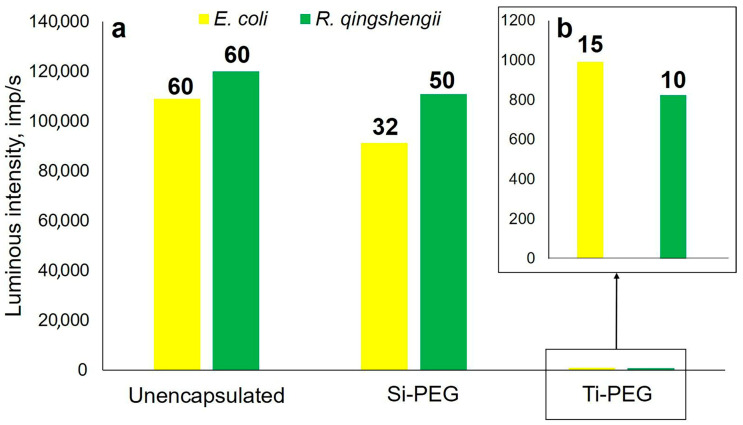
A diagram of the luminescence intensity of free and encapsulated microorganisms, with encapsulation using (**a**) Si-PEG and (**b**) Ti-PEG. The time of luminescence for each sample is indicated above the corresponding column on the diagram.

For free microorganisms and biohybrid materials based on them, two parameters were recorded: the intensity and the duration of glow (Figure 10). The analysis was performed until the luminescence intensity reached the level of the control sample. Free and encapsulated cells do not emit light without the addition of specific reagents, so the results are not affected by additional fluorescence.

Free microorganisms are characterized by the highest intensity and duration of luminescence, which is due to a large number of living cells and unhindered extraction of ATP molecules. Biohybrid materials based on Si-PEG show a decrease in luminescence intensity by 7% for *R. qingshengii* and 16% for *E. coli*. The glow time remains almost unchanged for *R. qingshengii*, but decreases by two times for *E. coli*. For biohybrids based on titanium compounds, the luminescent intensity was 5% that of free cells, and the luminescent time was 10 and 15 min for *R. qingshengii* and *E. coli*, respectively. These results support the toxic effects of Ti–O–, which is formed in the sol–gel process. As a result, microorganisms are in a state of oxidative stress, leading to the generation of intracellular reactive oxygen species. These species cause destructive effects within the cell, including the oxidation of coenzyme A and the peroxidation of lipids [63]. This leads to a decrease in respiratory activity and, ultimately, cell death caused by free radicals.

**Figure 11:**
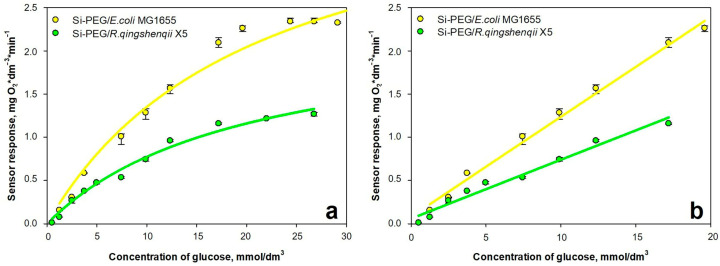
The dependence of the sensor responses vs. glucose concentration in the analytical cell: (**a**) the calibration dependence; (**b**) the linear segment.

Respiratory activity was studied for *E. coli* MG1655 and *R. qingshengii* X5, encapsulated only in Si-PEG-based hydrogels. The assessment was carried out using a biosensor approach, applying a Clark-type oxygen electrode, in accordance with the procedures described in Section 4.8.2, similar to those outlined in [18,25,64]. To determine the analytical properties of the biosensor, calibration curves were generated to illustrate the relationship between the sensor’s response and the glucose concentration in the analytical cell, which were then approximated to a linear segment (with an upper limit of *K_m_*), where the slope of the curve represents the sensitivity coefficient of the biosensor (Figure 11).

The sensitivity and stability of biosensors based on the biocatalysts are presented in Table 4.

It has been shown that biocatalysts can detect glucose concentration over a wide range for up to 10 days. Biosensors are characterized by stable operation, which is essential when developing various biosensitive surfaces and purification systems for aquatic ecosystems.

## 3. Conclusions

Hydrogels based on silicon and titanium polyolate have been used for encapsulation of individual cells for the first time in this article. The Si-PEG-based hydrogel has significant advantages over Ti-PEG-based hydrogel for bacterial encapsulation. It achieves higher encapsulation efficiency, with more than 70% compared to 50% for Ti-PEG, and preserves the viability of microorganisms without showing toxicity, as demonstrated by the absence of growth inhibition zones using the agar diffusion method. In contrast, Ti-PEG-based hydrogel reduces cell survival by 50%. The antibacterial action of Ti–O– is exerted through two processes. Firstly, its positively charged surface, when attached to the cell wall, can impair the protective function of the cell membrane. Simultaneously, microorganisms have oxidative stress, resulting in the production of intracellular reactive oxygen species. This results in a decline in respiratory activity and ultimately leads to cell death. Therefore, it has been demonstrated that the antimicrobial properties of titanium-containing materials are not always dependent on their photocatalytic activity. However, it is precisely this unique combination of properties—the presence of an inorganic mesh that is not photocatalytically active, combined with a predominant organic phase (PEG), which is biocompatible—that opens up an equally important niche for Ti-PEG-based hydrogel. We propose that such a system could become an excellent platform for enzyme immobilization. Enzymes, in contrast to whole cells, do not need high viability and complex metabolic processes. At the same time, the organic phase of PEG creates a favorable microenvironment for protein molecules, while the inorganic mesh provides mechanical stability. The mild antimicrobial activity of the hydrogel might even be beneficial, preventing microbial contamination and prolonging the service life of the enzymatic biocatalyst.

Si-PEG-based hydrogel creates an optimal “cell-in-shell” structure, confirmed by SEM. This makes Si-PEG based hydrogel a preferred choice for biohybrid materials, as it combines high biocompatibility, effective encapsulation and an optimal morphology, suitable for various biotechnological, environmental, and medical applications.

## 4. Materials and Methods

Bacteria *Escherichia coli* MG1655 and *Rhodococcus qingshengii* X5 (VKM Ac-2532D) were obtained from the All-Russian Collection of Microorganisms Federal Research Center Pushchino Scientific Center for Biological Research of The Russian Academy of Sciences’ G. K. Skryabin Institute of Biochemistry and Physiology of Microorganisms (Pushchino, Russia).

Silicon tetra(polyethylene glycolate) (Si-PEG) and titanium tetra(polyethylene glycolate) in 10 mol excess of poly(ethylene glycol) (Ti-PEG) were obtained from a research team led by T.G. Khonina at the I.Ya. Postovsky Institute of Organic Synthesis (Yekaterinburg, Russia) [43].

### 4.1. Cell Cultivation

Bacterial cells of *R. qingshengii* X5 (VKM Ac-2532D) and *E. coli* MG1655 were cultured on lysogenic nutrient medium LB under aeration at 180 rpm and at temperatures of 28 °C and 37 °C, respectively. The biomass was separated from the culture medium using an MPW 351R centrifuge (MPW Medical Instruments, Warsaw, Poland) at room temperature for 10 min at 7000× *g*. The supernatant was then rinsed with a saline solution (0.9% NaCl) and centrifuged again for 10 min at 7000× *g*. This procedure was repeated. The bacterial biomass was then stored at −20 °C in Eppendorf type tubes.

### 4.2. Synthesis Hydrogels and Biohybrid Materials Based on Silicon and Titanium Polyolate Compounds

To synthesize hydrogels from Si-PEG or Ti-PEG (Yekaterinburg, Russia), a 0.5 cm^3^ saline solution (0.9% NaCl, pharmaceutical grade, Solopharm, St. Petersburg, Russia) was added to 1.0 cm^3^ of the precursor (Si-PEG or Ti-PEG) and stirred on Vortex 2 (IKA, Staufen, Germany) for 5 min. Then, 0.025 cm^3^ of the nucleophilic catalyst solution of 0.2 mol/dm^3^ NaF (≥99.0%, Sigma-Aldrich, St. Louis, MO, USA) was added and stirred for 15 min. To form the polymer hydrogel, this material was left for 24 h at a temperature of +4 °C. The volume ratio H_2_O:Si- or Ti-PEG:NaF is 9:20:0.5.

To obtain biohybrid materials, a 0.125 cm^3^ bacterial suspension (*E. coli* MG1655 (1.0 ± 0.1) × 10^10^ CFU/cm^3^, *R. qingshengii* X5 (3 ± 1) × 10^9^ CFU/cm^3^) in saline solution (0.9% NaCl, pharmaceutical grade, Solopharm, St. Petersburg, Russia) was added to 0.25 cm^3^ polyolate components (Si-PEG or Ti-PEG, Yekaterinburg, Russia) and mixed for 5 min. Then, 0.025 cm^3^ of the nucleophilic catalyst solution of 0.2 mol/dm^3^ NaF (≥99.0%, Sigma-Aldrich, St. Louis, MO, USA) was added and stirred for 15 min. To form the polymer hydrogel, this material was left for 24 h at a temperature of +4 °C. As a result, biohybrid materials were obtained in the form of hydrogels.

### 4.3. Vibrational Spectroscopy and Thermogravimetric Analysis of Hydrogels

The structure and characteristic groups of the Ti-PEG-based hydrogel were confirmed using vibrational spectroscopy—FTIR- and Raman spectroscopy. An IR spectrometer, the INFRARED Fourier spectrometer Infralume FT-08 (Lumex, St. Petersburg, Russia), was used to record the IR spectra of the hydrogel in the wavelength range from 400 to 4000 cm^−1^ with a resolution of 4 cm^−1^. Raman spectra were also recorded for Si-PEG and Ti-PEG-based hydrogels using a Raman microscope M532 (InSpectr, St. Petersburg, Russia) at a magnification of 100 times and a 532 nm laser, in the wavelength range between 300 and 4000 cm^−1^. Accumulation time was 10 s and the number of averaged spectra was 2000.

The stability and chemical transformations of hydrogels based on polyethylene glycolates of silicon and titanium were studied in the temperature range 0–600 °C. Before analysis, the samples were dried at 40 °C to a constant weight. They were then placed on thermal weights TG 209 F1 (NETZSCH, Selb, Germany), and a treatment program was executed: the samples were heated at 60 °C (20 min), heated at a rate of 10 °C/min to 320 °C, held at that temperature for 10 min, the temperature was increased at the same rate to 390 °C (held for 10 min), and finally heated to 600 °C.

### 4.4. Photocatalytic Activity of the Ti-PEG-Based Hydrogel

The photocatalytic activity of titanium polyethylene glycol-based hydrogels was evaluated through the photodegradation of methylene blue (MB) under UV radiation. A typical experiment involved placing an aqueous solution of MB (1 mg/mL, 50 mL) and a hydrogel (0.1 g) in a reactor at 25 °C with constant stirring. The reaction vessel was located 10 cm away from a UV lamp (26 watts, 365 nm) as the light source. The reaction solution was stirred for 1 h in the dark to allow for adsorption equilibrium to be achieved. The photocatalytic reaction was then initiated by turning on a UV lamp. The suspension solution was centrifuged (MPW 351R, MPW Med. Instruments, Warsaw, Poland). The optical density of the solution was then measured and analyzed using an SF-2000 spectrophotometer (Spektr Design, Moscow, Russia) at an operating wavelength of 663 nm.

### 4.5. Effectiveness of Bacterial Encapsulation

Serial dilutions of the samples were prepared in saline solution. Free cell cultures and cell suspensions that had been previously washed off the hydrogel with saline solution were sown onto LB medium. All the seeded plates with nutrient agar were then incubated in a thermostat for 2 days.

### 4.6. Evaluation of the Viability of Encapsulated Bacteria

#### 4.6.1. Diffusion in Agar Method

To assess the sensitivity of bacteria to the initial components for their immobilization, dishes with LB medium were seeded with an inoculum of microorganisms. Then, 25 µL of saline solution (0.9% NaCl), NaF solution (0.2 mol/dm^3^), initial precursors (Si-PEG and Ti-PEG) and hydrogels based on them (obtained a day before seeding) were applied to the seeded, dried surface of the agar. The cups with microorganisms were incubated in a thermostat for 2 days.

#### 4.6.2. Fluorescence Microscopy

Live and dead cells were identified using the LIVE/DEAD BacLight Bacterial Viability Kit from Thermo Fisher Scientific (Waltham, MA, USA). A total of 0.001 cm^3^ of the corresponding dye from the kit was added to 0.5 cm^3^ of microbial suspension. Gels with a volume of approximately 0.05 cm^3^ were carefully inserted into a 0.5 cm^3^ of HEPES, and 0.001 cm^3^ of dye was added. The samples were then incubated for 15 min at 28 °C, and examined using fluorescence microscopy on an AXIO Imager A1 (Zeiss, Oberkochen, Germany), equipped with 56HE filters (Zeiss, Oberkochen, Germany) at a wavelength of 490 nm (excitation) and 512 nm + 630 nm (emission). Images were captured using an Axiocam 506 camera (Zeiss, Oberkochen, Germany).

### 4.7. Surface Morphology of the Biohybrid Materials—Immobilized Microorganisms in Si- and Ti-Polyolate-Based Hydrogels

The morphology of the biohybrid material surface was examined using scanning electron microscopy (SEM). A detailed description of the preparation of the sample for SEM is presented in [18].

### 4.8. Catalytic Activity of Immobilized Bacteria

#### 4.8.1. Bioluminescent Method for Determination of Intracellular ATP

AquaSnap Total rapid tests (Hygiena, Camarillo, CA, USA) were used to measure the bioluminescence of free and encapsulated microorganisms. The Biotox-10M device (NERA-S LLC, Moscow, Russia) was used to record the intensity of the signal in luminometer mode. For the experiment, a free culture was diluted 100 times and hydrogels containing encapsulated microorganisms were also diluted 10 times. They were then deposited on a vortex microcentrifuge CV-2500-IVD (Helikon, Moscow, Russia) and 0.8 cm^3^ of deionized water, and 0.2 cm^3^ of each sample were taken for analysis. The bioluminescence of the solution was measured under irradiation at a wavelength range of 300–600 nm. A reagent was then added to extract ATP from cells and initiate a bioluminescent reaction, and the signal was recorded until it reached a steady state. The maximum bioluminescence and glow time of each sample was then recorded.

#### 4.8.2. Biosensor Approach

To determine the respiratory activity of bacteria, a working bioreceptor element was created. A total of 0.05 mL of biohybrid materials was applied to the surface of an oxygen electrode and covered with a nylon mesh. The respiratory activity was measured using the dissolved oxygen analyzer “Expert-009” (Econix Expert, Moscow, Russia) in “thermo-oximeter” mode, as detailed in [18]. The results were statistically processed using SigmaPlot ver. 12.5 software [65].

## Figures and Tables

**Table 1:** Determination the number of CFUs for cells before and after encapsulation.

Microorganisms	Control, CFU/mL	Si-PEG, CFU/mL	EE (Si-PEG), %	Ti-PEG, CFU/ml	EE (Ti-PEG), %
*E coli* MG1655	(1.0 ± 0.1) × 10^10^	(2 ± 1) × 10^7^	72	(3 ± 1) × 10^5^	54
*R. qingshengii* X5	(3 ± 1) × 10^9^	(2 ± 1) × 10^7^	77	(4 ± 2) × 10^4^	50

**Table 2:** Comparative analysis of the obtained results with previous studies.

Bacteria	Matrices	EE, %	Ref.
*E coli* MG1655	Si-PEG-based hydrogel	72	This work
	Ti-PEG-based hydrogel	54	
*R. qingshengii* X5	Si-PEG-based hydrogel	77	This work
	Ti-PEG-based hydrogel	50	
*L. brevis C23*	*S. cerevisiae* cell wall	90	[53]
	Sodium alginate	74	
*L. plantarum* K014	*S. cerevisiae* cell wall	83	
	Sodium alginate	69	
*R*. *palustris* KTSSR54	Sodium alginate	51	[54]
	2% Sodium alginate + 4% Starch	71	
*P. fluorescens*	3% Chitosan	67	[55]
*L. bulgaricus*	Carrageenan-alginate (extrusion)	34–43	[56]

**Table 3:** The zones of inhibition of microbial growth, determined by the agar diffusion method.

Components	Lysis Zones, mm	
	*E. coli* MG1655	*R. qingshengii* X5
NaCl	―	―
NaF	―	―
Precursor Si-PEG	―	―
Hydrogel based on Si-PEG	―	―
Precursor Ti-PEG	10 ± 1	8 ± 1
Hydrogel based on Ti-PEG	8.5 ± 0.3	7.0 ± 0.1

**Table 4:** The sensitivity and stability of biosensors.

Parameter	Si-PEG/*E. coli* MG1655	Si-PEG/*R. qingshengii* X5
The range of detectable concentration, mmol/dm^3^	1–21	1–20
Sensitivity coefficient, mgO_2_ × min^−1^ × mmol^−1^	0.116 ± 0.002	0.068 ± 0.002
Relative standard deviation, %	13	4
Long-term stability, days	9	8

## Data Availability

Dataset available on request from the authors.

## Abbreviations

Si-PEG	Silica-polyethylene glycol
Ti-PEG	Titanium-polyethylene glycol
PEG	Polyethylene glycol
PVA	Polyvinyl alcohol
ORMOSIL	Organically modified silica
TEOS	Tetraethylorthosilicate
TG	Thermogram
DTG	Differential thermogram
MB	Methylene blue
TMOS	Tetramethylorthosilicate
TBOT	Tetrabutoxytitanium
ATP	Adenosine triphosphate
